# Construction and validation of a predictive model for fear of progression among postoperative patients with breast cancer: a multi-center cross-sectional study

**DOI:** 10.1186/s12885-026-16468-3

**Published:** 2026-07-27

**Authors:** Lingyun Tian, Xinyu Feng, Mengyuan Liu, Jing Jiang, Qi Qin, Wenli Xie, Yang Guo, Jing Zhao, Yu Liang, Xiaopeng Ma, Lin Jiang

**Affiliations:** 1 Department of Health Management Center, Division of Life Sciences and Medicine, the First Affiliated Hospital of USTC, University of Science and Technology of China, Hefei, Anhui China; 2 Department of Nursing, Division of Life Sciences and Medicine, the First Affiliated Hospital of USTC, University of Science and Technology of China, Hefei, Anhui China; 3 School of Nursing, The Hong Kong Polytechnic University, Hung Hom, Kowloon, Hong Kong SAR China; 4 Xiangya School of Nursing, Central South University, Changsha, Hunan China; 5 School of Nursing, Bengbu Medical University, Bengbu, Anhui China; 6 Department of General Surgery, Division of Life Science and Medicine, The First Affiliated Hospital of USTC, University of Science and Technology of China, Hefei, Anhui China

**Keywords:** Fear of progression, Postoperative patients, Breast cancer, Predictive model, Nomogram

## Abstract

**Background:**

Fear of progression (FoP) is a prevalent psychological response to the real threats associated with cancer diagnosis, treatment, and disease trajectory, and it represents one of the most prominent sources of distress symptoms among patients with breast cancer. Persistently elevated FoP may not only negatively impact treatment outcomes but also contribute to excessive healthcare utilization and reduced quality of life. This study aimed to construct and validate a predictive model for FoP in postoperative breast cancer patients based on the Health Ecology Model.

**Methods:**

This multi-center cross-sectional study enrolled 347 postoperative breast cancer patients from three tertiary Grade-A general hospitals in Anhui Province, China, between March 25, 2024, and June 25, 2024. Univariable analysis and multivariable logistic regression were employed to identify independent predictors of FoP. A nomogram was then constructed based on these predictors and internally validated. Model performance was assessed in terms of discrimination, calibration, and clinical usefulness using receiver operating characteristic (ROC) curves, calibration plots, and decision curve analysis (DCA), respectively.

**Results:**

The prevalence of high FoP was 52.74%. Multivariable logistic regression identified chemotherapy, frequency of physical activity, work status, sense of coherence, illness perception, and coping mode as independent predictors of FoP, which were incorporated into the nomogram. The model demonstrated excellent discriminatory power, with areas under the ROC curve (AUC) of 0.867 and 0.898 for the training and validation sets, respectively. Calibration plots indicated good agreement between predicted and observed outcomes, while DCA indicated favorable clinical utility across a range of threshold probabilities.

**Conclusions:**

A predictive model for FoP among postoperative breast cancer patients was successfully developed and validated within the HEM framework. This model may facilitate the early identification of individuals at high risk for FoP and provide a foundation for targeted interventions aimed at alleviating psychological distress and improving overall well-being.

## Introduction

According to the Global Cancer Statistics 2022, breast cancer was the most prevalent malignancy among women in 157 countries, with nearly 2.3 million new cases and 670,000 deaths reported globally [1]. In China, both the incidence and mortality of breast cancer have been rising steadily, with projections indicating rises of 11.1% and 43.7%, respectively, by 2045 [2]. While surgical resection is the cornerstone of breast cancer treatment, the risk of postoperative recurrence or metastasis remains considerable, necessitating adjunctive treatments including chemotherapy, radiotherapy, targeted therapy, and endocrine therapy combined to reduce the risk of disease recurrence [3]. However, these treatments may also impose substantial long-term physical and psychological burdens, including premature ovarian failure, infertility, body-image disturbances, altered sexual function, and ongoing worry about disease progression during recovery [4]. In this context, patients may develop persistent and amplified fears related to the realistic possibility of disease progression, which can significantly impair their well-being and quality of life [5].

Fear of progression (FoP) was defined by Dankert et al. [6] as the fear that an illness may progress or recur with its associated biological, psychological, and social consequences. In the context of cancer, patients face a persistent and tangible threat, which may generate substantial concern regarding disease progression and its biopsychosocial impact [7–11]. FoP differs from fear of recurrence in that the latter specifically refers to fear that cancer will return after remission, whereas FoP encompasses a broader and more continuous apprehension that the disease may deteriorate or advance at any stage of the illness trajectory [12]. Although FoP is generally considered as a natural and understandable response to the real threats associated with diagnosis, treatment, and uncertainty about prognosis, excessively high level of FoP can become maladaptive, affecting psychological well-being, social functioning, interpersonal relationships, and patients’ ability to cope effectively with the disease [13]. Moreover, heightened fear may undermine treatment adherence, potentially leading to suboptimal clinical outcomes and increased healthcare utilization [5]. Dinkel et al. [5]suggested that the vast majority of breast cancer patients experienced profound concerns, including fear of cancer spread, anxiety about intensive medical treatments and follow-up examination, and fear of pain. Importantly, growing evidence suggests that FoP can be effectively mitigated through targeted therapeutic interventions, such as mindfulness-based stress reduction (MBSR) program and cognitive behavior therapy (CBT) [14]. These findings underscore the critical significance of identifying individuals at risk for heightened FoP at an early stage, thereby enabling timely intervention to reduce psychological distress and promote efficient use of healthcare resources.

Previous studies have identified a variety of factors associated with FoP, including sociodemographic characteristics (e.g., age, region, education levels, marital status, residence) [15], clinical factors (e.g., tumor stage, illness status, cancer recurrence) [15, 16], and psychosocial factors (e.g., depression, anxiety, posttraumatic stress disorder, social support) [16, 17]. One study also found statistically significant correlations between FoP and intrusive thoughts, coping styles, and hyperarousal symptoms [18]. Additionally, inadequate health literacy and low self-efficacy have been linked to higher levels of FoP [19, 20]. However, these findings remain fragmented and is seldom integrated within a coherent theoretical framework, which limits the interpretability, predictive accuracy, and generalizability of current research. Theory-based predictive modeling is therefore needed to provide a more systematic understanding of the mechanisms underlying FoP and to improve the early identification of high-risk individuals. This effort may also inform the development of personalized, evidence-based interventions aimed at reducing FoP and improving overall survivorship outcomes.

The Health Ecology Model (HEM) proposed that individual health outcomes are shaped by five interconnected factors: individual traits, behavioral and psychological factors, interpersonal networks, living and working conditions, and the broader policy environment [21]. This holistic framework has been widely applied in research on disease prevention and health promotion, particularly among individuals with chronic diseases and other vulnerable populations, demonstrating strong utility in explaining and predicting health-related health outcomes [22, 23]. Nomograms are practical and reliable predictive tools that combine multiple risk variables into intuitive graphical representations, enabling the visualization of complex data and individualized assessment of clinical events. In the field of breast cancer, nomograms have been extensively used to predict recurrence, metastasis, treatment response, and survival [24, 25]. However, to the best of our knowledge, no previous study has systematically incorporated the HEM into the development of a nomogram-based model for predicting cancer-related psychological risk. Therefore, in this study, we used the HEM as the guiding theoretical framework to identify key factors associated with FoP among postoperative breast cancer patients. Based on these insights, we developed and validated a nomogram to predict the risk of FoP. This theory-informed predictive model may provide both a conceptual basis and a practical tool for the early identification of high-risk individuals, thereby supporting more targeted and personalized psychological care for patients with breast cancer.

## Methods

### Study design and participants

This study employed a multicenter cross-sectional design. Participants were recruited from March 25, 2024, to June 25, 2024, across three tertiary Grade-A general hospitals in Anhui Province, China. Individuals were included if they: (1) diagnosed with primary breast cancer according to the diagnostic criteria [26]; (2) had undergone surgical resection; (3) were aged > 18 years; (4) had no mental illness; (5) possessed adequate comprehension and expressive abilities; and (6) were informed and willing to participate in the study. The exclusion criteria were as follows: (1) patients with concurrent diagnosis of other malignant tumors; and (2) patients with cognitive impairment.

### Sample size

In this study, we identified 30 potential predictive factors through a comprehensive literature review and expert consultations to determine the necessary sample size for logistic analysis. Based on the recommendation of 5–10 cases per independent variable [27], and considering a 10% rate of invalid questionnaires and the reported incidence of FoP among chemotherapy-treated breast cancer patients in mainland China, which stands at 66.32% [28], the estimated sample size for the modeling group was calculated as follows:

$$\text{Sample Size} = \frac{30 \times 5}{66.32\% \times\left(1-10\%\right)}\approx252 \,\text {cases}$$

For the validation group, the sample size should be set between 25% and 50% of the modeling group [29], resulting in a minimum requirement of 63 cases.

### Measures

#### Predictive variables

**Fig. 1:**
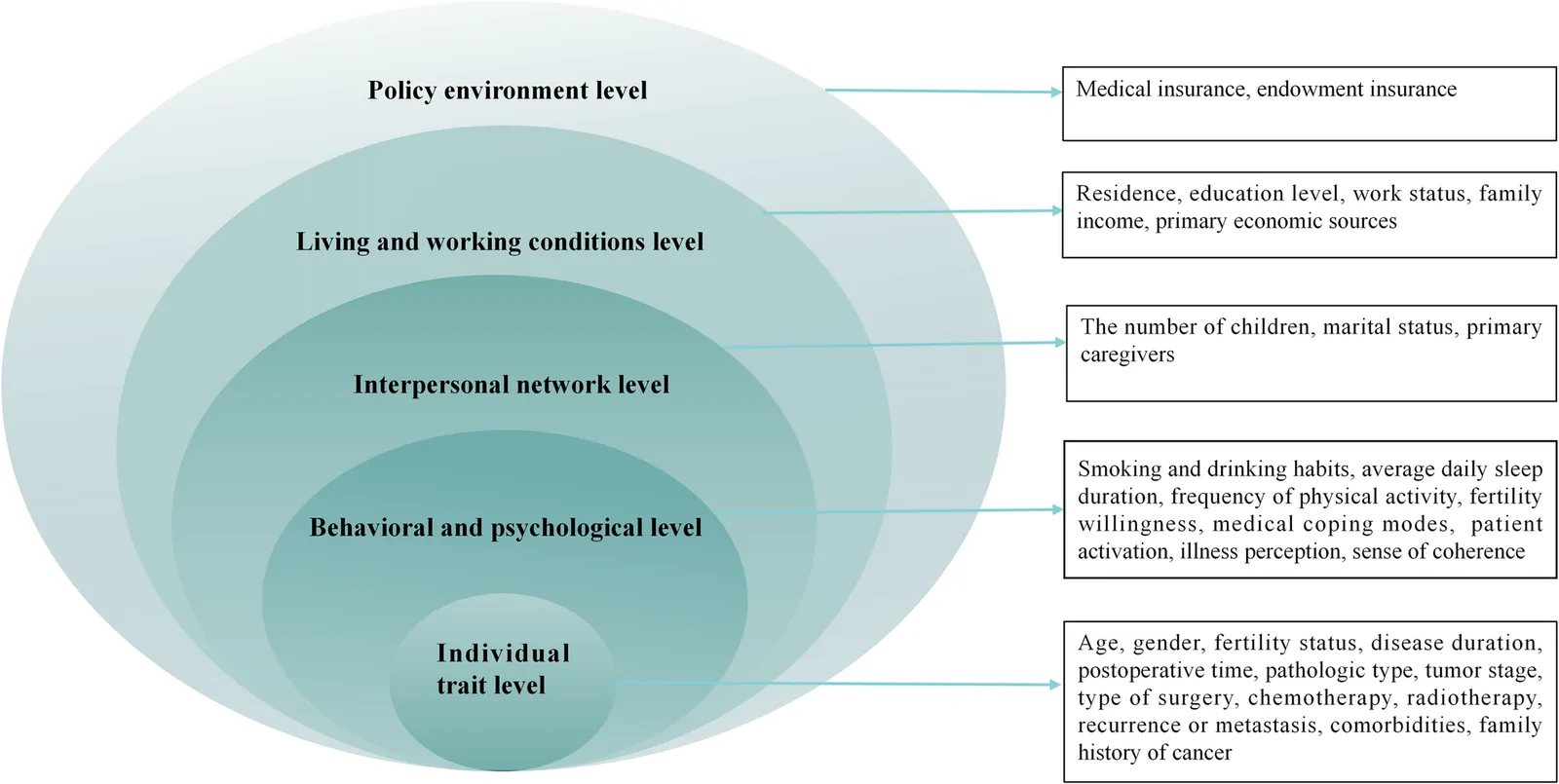
Categorization of variables according to the five-level framework of the HEM

Figure 1. presents the categorization of variables according to the five-level framework of the HEM.

##### Individual trait level

A self-designed questionnaire was used to collect data on age, gender, fertility status, disease duration, postoperative time, pathologic type, tumor stage, type of surgery, chemotherapy, radiotherapy, recurrence or metastasis, comorbidities, and family history of cancer.

##### Behavioral and psychological level

1. A self-designed questionnaire was used to collect data on smoking and drinking habits, average daily sleep duration, frequency of physical activity, and fertility willingness.
2. Medical Coping Modes Questionnaire (MCMQ)

The MCMQ, originally developed by Feifel et al. [30] and translated into Chinese by Shen et al. [31], evaluates patients’ coping strategies in response to illness. The scale comprises 20 items across three dimensions: confrontation (8 items), avoidance (7 items), and acceptance-resignation (5 items). Each item is rated on a 4-point Likert scale, with 8 items reverse-scored. The dimension with the highest cumulative score indicates the predominant coping strategy employed by the individual. In this study, the Cronbach’s α for the overall scale was 0.636.

(3)  The Patient Activation Measure-13 (PAM-13)

The PAM-13, initially developed by Hibbard et al. [32]and adapted into Chinese by Chen [33], is a 13-item scale assessing patient’ self-reported knowledge, skills, and confidence in managing their own health or chronic conditions. The total score ranges from 0 to 100, with higher scores reflecting greater confidence and capability in self-management behaviors and life-style modifications. In this study, the Cronbach’s α for the PAM-13 was 0.879.

(4) The Brief Illness Perception Questionnaire (BIPQ)

The BIPQ, created by Broadbent et al. [34] and translated into Chinese by Mei et al. [35], assesses individuals’ cognitive and emotional perceptions of their illness. The scale includes 9 items divided into three components: cognitive illness representations (5 items), emotional representations (2 items), and illness comprehensibility (1 item). Each item is rated on a 10-Likert scale, resulting in a total score ranging from 0 to 80. The higher scores indicated a stronger perception of illness threat and more negative emotional responses. In this study, the Cronbach’s α coefficient for the BIPQ was 0.627.

(5)  Sense of Coherence-13 (SOC-13)

The SOC-13, first proposed by Antonovsky [36] and translated into Chinese by Bao et al. [37], consists of 13 items distributed across three subdomains: comprehensibility, manageability, and meaningfulness. Each item is scored on a 7-point Likert scale, with 5 items reverse-scored. Total scores range from 13 to 91, where higher scores indicate a stronger sense of coherence. Based on score ranges, the levels of SOC are categorized as low (13–63), moderate (64–79), or high (80–91). The Cronbach’s α for this scale in the present study was 0.742.

##### Interpersonal network level

A self-designed questionnaire was used to collect data on the number of children, marital status, and primary caregivers.

##### Living and working conditions level

A self-designed questionnaire was used to collect data on residence, education level, work status, family income, and primary economic sources.

##### Policy environment level

A self-designed questionnaire was used to collect data on medical insurance and endowment insurance.

#### Fear of progression questionnaire-short form (FoP-Q-SF)

The FoP-Q-SF, developed by Mehnert et al. [16] and culturally adapted into Chinese by Wu et al. [38], evaluates the psychological burden of fear of disease progress. The 12-item questionnaire encompasses two dimensions: physical health and social-family concerns. Responses are rated on a 5-point Likert scale, yielding a total score ranging from 12 to 60. Higher scores indicate greater fear of progression, with a score of 34 or above serving as the threshold for identifying individuals with statistically significant psychological dysfunction related to FoP [39]. In this study, the instrument demonstrated a high internal consistency with a Cronbach’s α of 0.887.

### Data collection

In this study, data collection was conducted using an online questionnaire platform in China to enhance both convenience and efficiency. Prior to the survey, the research team established collaborations with the heads of breast surgery departments at three hospitals, to ensure that they were informed about the study’s objectives, procedures, and key considerations. Eligible participants were recruited based on predefined inclusion and exclusion criteria between March 25 and June 25, 2024. Upon obtaining informed consent, participants received a link to the questionnaire via WeChat. The questionnaire included step-by-step instructions to ensure accurate and genuine responses, and all questions were mandatory to minimize missing data. Responses were considered valid once participants completed all questions and confirmed their submissions. Privacy was safeguarded through anonymous submissions, and we assured participants that all collected data would remain strictly confidential and used solely for scientific research purposes. Participation was entirely voluntary, and participants were informed of their right to withdraw from the study at any time without providing a reason. Throughout the research process, no harm or adverse effects were caused to participants. Ultimately, 351 individuals responded to the questionnaire, with 4 opting not to complete it, resulting in a final sample size of 347 valid responses.

### Statistical analysis

Statistical analysis was performed using SPSS 26.0 and R version 4.1.3 software. Mean ± SD was employed to present continuous variables with a normal distribution, while median (P25, P75) was used for those with a non-normal distribution. Categorical data were presented as counts or percentages. The predictive variables of FoP in postoperative patients with breast cancer were firstly analyzed using univariable analysis: Two independent sample t-tests were employed to compare normally distributed data between two groups. The non-parametric Mann-Whitney U tests were employed to process the non-normally distributed data. The comparison of categorical data between the two groups was conducted using the Pearson chi-square test or Fisher’s exact test. Variables with *P* < 0.05 in the univariable analysis were incorporated into the multivariable logistic regression analysis. To identify and choose the final predictive variables for inclusion in the model, a backward (conditional) multivariable logistic regression was conducted using the Akaike Information Criterion as a screening tool [40, 41]. The glm package in R was utilized to perform multivariable logistic regression analysis. Subsequently, the rms package in R was employed to establish a nomogram model based on the multivariable model. The discrimination of the proposed nomogram model was assessed by the area under the curve (AUC) using the pROC, ggROC, and fbroc packages in the R language. The calibration was evaluated through both Hosmer-Lemeshow goodness-of-fit test and visual inspection using the calibrate function in the rms package in R, as well as the val. prob and HL test packages. Furthermore, to determine the clinical utility of the model, decision curve analysis (DCA) was conducted using rmda and dcurves packages in R to evaluate its clinical effectiveness.

## Results

### Participant characteristics

A total of 347 postoperative patients with breast cancer were recruited in this study. Table 1 showed the baseline characteristics of participants. Among them, the age of the participants ranged from 22 to 76 years (50.71 ± 9.74). The average score of FoP were (33.78 ± 9.50). Participants with a score ≥ 34 was classified into high level group (*n* = 183), otherwise they were classified into low level group (*n* = 164). The rate of psychological dysfunction was 52.74% (183/347). Based on an 8:2 ratio, we randomly divided these patients into two groups: a training set (*n* = 277) used to establish the predictive model and a validation set (*n* = 70) utilized to access the model’s performance. The results showed that there were no statistically significant differences in any variables between the two groups (*P* > 0.05), and the data was comparable.

**Table 1:** The baseline characteristics of the enrolled patients in the training and validation sets

Variables	Total	Training	Validation	Statistic	*P*
	*n* = 347	*n* = 277	*n* = 70		
Age (y), Median (Q1, Q3)	52 (45, 57)	52 (45, 57)	53 (46.25, 57)	9037.5	0.381
Gender				Fisher	0.492
Male	3 (0.9%)	2 (0.7%)	1 (1.4%)		
Female	344 (99.1%)	275 (99.3%)	69 (98.6%)		
Fertility status				Fisher	1.000
No	7 (2.0%)	6 (2.2%)	1 (1.4%)		
Yes	340 (98.0%)	271 (97.8%)	69 (98.6%)		
Disease duration (months), Median (Q1, Q3)	7 (3, 14)	7 (3, 14)	7 (3, 12.75)	9757.5	0.934
Postoperative time (months), Median (Q1, Q3)	6 (2, 12)	6 (2, 12)	5.5 (1.87, 12.5)	9745	0.947
Pathologic type				1.917	0.590
Non-invasive breast cancer	27 (7.8%)	21 (7.6%)	6 (8.6%)		
Early-stage invasive breast cancer	122 (35.2%)	93 (33.6%)	29 (41.4%)		
Special types of invasive breast cancer	32 (9.2%)	27 (9.7%)	5 (7.1%)		
Invasive breast cancer	166 (47.8%)	136 (49.1%)	30 (42.9%)		
Tumor stage				Fisher	0.225
1	59 (17.0%)	47 (17.0%)	12 (17.1%)		
2	184 (53.0%)	140 (50.5%)	44 (62.9%)		
3	86 (24.8%)	74 (26.7%)	12 (17.1%)		
4	18 (5.2%)	16 (5.8%)	2 (2.9%)		
Type of surgery				3.376	0.185
Breast-conserving surgery	81 (23.3%)	65 (23.5%)	16 (22.9%)		
Mastectomy	238 (68.6%)	186 (67.1%)	52 (74.3%)		
Mastectomy and breast reconstruction	28 (8.1%)	26 (9.4%)	2 (2.9%)		
Chemotherapy				0.335	0.562
No	39 (11.2%)	33 (11.9%)	6 (8.6%)		
Yes	308 (88.8%)	244 (88.1%)	64 (91.4%)		
Radiotherapy				0.04	0.841
No	217 (62.5%)	172 (62.1%)	45 (64.3%)		
Yes	130 (37.5%)	105 (37.9%)	25 (35.7%)		
Recurrence or metastasis				0.102	0.749
No	306 (88.2%)	243 (87.7%)	63 (90.0%)		
Yes	41 (11.8%)	34 (12.3%)	7 (10.0%)		
Comorbidities				0.848	0.357
No	287 (82.7%)	226 (81.6%)	61 (87.1%)		
Yes	60 (17.3%)	51 (18.4%)	9 (12.9%)		
Family history of cancer				0.033	0.857
No	307 (88.5%)	246 (88.8%)	61 (87.1%)		
Yes	40 (11.5%)	31 (11.2%)	9 (12.9%)		
Smoking				Fisher	0.571
Never	337 (97.1%)	270 (97.5%)	67 (95.7%)		
Quitted	6 (1.7%)	4 (1.4%)	2 (2.9%)		
Yes	4 (1.2%)	3 (1.1%)	1 (1.4%)		
Drinking				Fisher	0.080
Never	283 (81.6%)	221 (79.8%)	62 (88.6%)		
Quitted	62 (17.9%)	55 (19.9%)	7 (10.0%)		
Yes	2 (0.6%)	1 (0.4%)	1 (1.4%)		
An average daily sleep duration of 6–8 h				0.136	0.713
No	118 (34.0%)	96 (34.7%)	22 (31.4%)		
Yes	229 (66.0%)	181 (65.3%)	48 (68.6%)		
Frequency of physical activity				7.575	0.023
Never	123 (35.4%)	102 (36.8%)	21 (30.0%)		
<3 times/week	145 (41.8%)	106 (38.3%)	39 (55.7%)		
≥ 3 times/week	79 (22.8%)	69 (24.9%)	10 (14.3%)		
Fertility willingness				0.732	0.392
Yes	59 (17.0%)	50 (18.1%)	9 (12.9%)		
No	288 (83.0%)	227 (81.9%)	61 (87.1%)		
Number of children				Fisher	0.760
0	5 (1.4%)	4 (1.4%)	1 (1.4%)		
1	165 (47.6%)	129 (46.6%)	36 (51.4%)		
≥ 2	177 (51.0%)	144 (52.0%)	33 (47.1%)		
Marital status				Fisher	0.782
Unmarried	4 (1.1%)	4 (1.4%)	0 (0.0%)		
Married	324 (93.4%)	258 (93.1%)	66 (94.3%)		
Divorced	8 (2.3%)	7 (2.5%)	1 (1.4%)		
Widowed	11 (3.2%)	8 (2.9%)	3 (4.3%)		
Primary caregivers				Fisher	0.990
Parents	34 (9.8%)	27 (9.7%)	7 (10.0%)		
Partner	215 (62.0%)	172 (62.1%)	43 (61.4%)		
Offspring	80 (23.1%)	64 (23.1%)	16 (22.9%)		
Other relatives	18 (5.2%)	14 (5.1%)	4 (5.7%)		
Residence				< 0.001	0.999
Rural	176 (50.7%)	141 (50.9%)	35 (50.0%)		
Urban	171 (49.3%)	136 (49.1%)	35 (50.0%)		
Education level				1.933	0.586
Elementary school or below	117 (33.7%)	90 (32.5%)	27 (38.6%)		
Middle school	119 (34.3%)	99 (35.7%)	20 (28.6%)		
High school	60 (17.3%)	49 (17.7%)	11 (15.7%)		
University or above	51 (14.7%)	39 (14.1%)	12 (17.1%)		
Work status				1.243	0.537
Be on the job	92 (26.5%)	76 (27.4%)	16 (22.9%)		
Unemployed	181 (52.2%)	145 (52.3%)	36 (51.4%)		
Retired	74 (21.3%)	56 (20.2%)	18 (25.7%)		
Family income (per capita monthly, yuan)				4.506	0.212
<2000	73 (21.0%)	63 (22.7%)	10 (14.3%)		
2000 ~ 4000	122 (35.2%)	93 (33.6%)	29 (41.4%)		
4001 ~ 6000	84 (24.2%)	70 (25.3%)	14 (20.0%)		
>6000	68 (19.6%)	51 (18.4%)	17 (24.3%)		
Primary economic sources				0.372	0.830
Pension	71 (20.5%)	56 (20.2%)	15 (21.4%)		
Financial assistance provided by family and friends	84 (24.2%)	69 (24.9%)	15 (21.4%)		
Labor income	192 (55.3%)	152 (54.9%)	40 (57.1%)		
Medical insurance				Fisher	0.139
Yes	328 (94.5%)	259 (93.5%)	69 (98.6%)		
No	19 (5.5%)	18 (6.5%)	1 (1.4%)		
Endowment insurance				0.152	0.697
Yes	198 (57.1%)	160 (57.8%)	38 (54.3%)		
No	149 (42.9%)	117 (42.2%)	32 (45.7%)		
Total score of sense of coherence, Median (Q1, Q3)	56 (49, 64)	56 (49, 63)	57 (51, 66.75)	8648	0.163
Total score of illness perception, Median (Q1, Q3)	41 (34, 47)	41 (34, 47)	41 (33, 45)	10,191	0.508
Confrontation, Median (Q1, Q3)	20 (17, 22)	19 (17, 22)	20 (18, 22)	9401	0.694
Avoidance, Median (Q1, Q3)	16 (14, 18)	16 (14, 18)	16 (14, 17)	9570.5	0.868
Acceptance-resignation, Median (Q1, Q3)	9 (6, 10)	9 (6, 10)	8 (6, 10)	9939	0.743
Total score of positivity, Median (Q1, Q3)	39 (37, 45)	39 (37, 45)	39 (37, 44)	10,214	0.487
Average Score of FoP, Mean ± SD	33.78 ± 9.5	33.9 ± 9.53	33.31 ± 9.46	0.458	0.648
Psychological dysfunction, n (%)				0.144	0.704
No	164 (47.3%)	129 (46.6%)	35 (50.0%)		
Yes	183 (52.7%)	148 (53.4%)	35 (50.0%)		

### Construction of the nomogram

Univariate analysis was performed to examine the potential risk factors associated with psychological dysfunction of participants in the training set. The outcomes of the univariable analysis were presented in Table 2. Variables that exhibited a significance level of *P* < 0.05 during the univariable analysis included age, disease duration, postoperative time, chemotherapy, frequency of physical activity, residence, work status, primary economic sources, sense of coherence, disease perception, and coping mode.

**Table 2:** Results of univariable analysis for fop in the training set

Variables	Total	Low-level group	High-level group	Statistic	*P*
	*n* = 277	*n* = 129	*n* = 148		
Age (y), Median (Q1, Q3)	52 (45, 57)	53 (46, 58)	50.5 (43, 56)	11,125	*0.018*
Gender				Fisher	1.000
Male	2 (0.7%)	1 (0.8%)	1 (0.7%)		
Female	275 (99.3%)	128 (99.2%)	147 (99.3%)		
Fertility status				Fisher	1.000
No	6 (2.2%)	3 (2.3%)	3 (2.0%)		
Yes	271 (97.8%)	126 (97.7%)	145 (98.0%)		
Disease duration, Median (Q1, Q3)	7 (3, 14)	10 (4, 18)	6 (2, 12)	11,515	*0.003*
Postoperative time, Median (Q1, Q3)	6 (2, 12)	7 (3, 15)	5 (1.38, 9)	11409.5	*0.005*
Pathologic type				2.147	0.543
Non-invasive breast cancer	21 (7.6%)	7 (5.4%)	14 (9.5%)		
Early-stage invasive breast cancer	93 (33.6%)	46 (35.7%)	47 (31.8%)		
Special types of invasive breast cancer	27 (9.7%)	14 (10.9%)	13 (8.8%)		
Invasive breast cancer	136 (49.1%)	62 (48.1%)	74 (50.0%)		
Tumor stage				0.564	0.905
1	47 (17.0%)	22 (17.1%)	25 (16.9%)		
2	140 (50.5%)	66 (51.2%)	74 (50.0%)		
3	74 (26.7%)	35 (27.1%)	39 (26.4%)		
4	16 (5.8%)	6 (4.7%)	10 (6.8%)		
Type of surgery				0.143	0.931
Breast-conserving surgery	65 (23.5%)	29 (22.5%)	36 (24.3%)		
mastectomy	186 (67.1%)	88 (68.2%)	98 (66.2%)		
Mastectomy and breast reconstruction	26 (9.4%)	12 (9.3%)	14 (9.5%)		
Chemotherapy				6.522	*0.011*
No	33 (11.9%)	8 (6.2%)	25 (16.9%)		
Yes	244 (88.1%)	121 (93.8%)	123 (83.1%)		
Radiotherapy				0.121	0.728
No	172 (62.1%)	82 (63.6%)	90 (60.8%)		
Yes	105 (37.9%)	47 (36.4%)	58 (39.2%)		
Recurrence or metastasis				1.498	0.221
No	243 (87.7%)	117 (90.7%)	126 (85.1%)		
Yes	34 (12.3%)	12 (9.3%)	22 (14.9%)		
Comorbidities				0.054	0.816
No	226 (81.6%)	104 (80.6%)	122 (82.4%)		
Yes	51 (18.4%)	25 (19.4%)	26 (17.6%)		
Family history of cancer				1.37	0.242
No	246 (88.8%)	111 (86.0%)	135 (91.2%)		
Yes	31 (11.2%)	18 (14.0%)	13 (8.8%)		
Smoking				Fisher	0.087
Never	270 (97.5%)	123 (95.3%)	147 (99.3%)		
Quitted	4 (1.4%)	3 (2.3%)	1 (0.7%)		
Yes	3 (1.1%)	3 (2.3%)	0 (0.0%)		
Drinking				Fisher	0.597
Never	221 (79.8%)	101 (78.3%)	120 (81.1%)		
Quitted	55 (19.9%)	28 (21.7%)	27 (18.2%)		
Yes	1 (0.4%)	0 (0.0%)	1 (0.7%)		
Average daily sleep duration of 6–8 h				0.921	0.337
No	96 (34.7%)	49 (38.0%)	47 (31.8%)		
Yes	181 (65.3%)	80 (62.0%)	101 (68.2%)		
Frequency of physical activity				22.356	*< 0.001*
Never	102 (36.8%)	32 (24.8%)	70 (47.3%)		
< 3 times/week	106 (38.3%)	50 (38.8%)	56 (37.8%)		
≥ 3 times/week	69 (24.9%)	47 (36.4%)	22 (14.9%)		
Fertility willingness				1.405	0.236
Yes	50 (18.1%)	19 (14.7%)	31 (20.9%)		
No	227 (81.9%)	110 (85.3%)	117 (79.1%)		
Number of children				Fisher	0.829
0	4 (1.4%)	2 (1.6%)	2 (1.4%)		
1	129 (46.6%)	63 (48.8%)	66 (44.6%)		
≥ 2	144 (52.0%)	64 (49.6%)	80 (54.1%)		
Marital status				Fisher	0.461
Unmarried	4 (1.4%)	3 (2.3%)	1 (0.7%)		
Married	258 (93.1%)	117 (90.7%)	141 (95.3%)		
Divorced	7 (2.5%)	4 (3.1%)	3 (2.0%)		
Widowed	8 (2.9%)	5 (3.9%)	3 (2.0%)		
Primary caregivers				5.183	0.159
Parents	27 (9.7%)	8 (6.2%)	19 (12.8%)		
Partner	172 (62.1%)	80 (62.0%)	92 (62.2%)		
Offspring	64 (23.1%)	32 (24.8%)	32 (21.6%)		
Other relatives	14 (5.1%)	9 (7.0%)	5 (3.4%)		
Residence				8.59	*0.003*
Rural	141 (50.9%)	53 (41.1%)	88 (59.5%)		
Urban	136 (49.1%)	76 (58.9%)	60 (40.5%)		
Education level				2.629	0.452
Elementary school or below	90 (32.5%)	40 (31.0%)	50 (33.8%)		
Middle school	99 (35.7%)	42 (32.6%)	57 (38.5%)		
High school	49 (17.7%)	27 (20.9%)	22 (14.9%)		
University or above	39 (14.1%)	20 (15.5%)	19 (12.8%)		
Work status, n (%)				25.768	*< 0.001*
Be on the job	76 (27.4%)	30 (23.3%)	46 (31.1%)		
Unemployed	145 (52.3%)	56 (43.4%)	89 (60.1%)		
Retired	56 (20.2%)	43 (33.3%)	13 (8.8%)		
Family income (per capita monthly, yuan)				6.33	0.097
<2000	63 (22.7%)	30 (23.3%)	33 (22.3%)		
2000 ~ 4000	93 (33.6%)	50 (38.8%)	43 (29.1%)		
4001 ~ 6000	70 (25.3%)	24 (18.6%)	46 (31.1%)		
>6000	51 (18.4%)	25 (19.4%)	26 (17.6%)		
Primary economic sources				21.721	*< 0.001*
Pension	56 (20.2%)	41 (31.8%)	15 (10.1%)		
Financial assistance provided by family and friends	69 (24.9%)	23 (17.8%)	46 (31.1%)		
Labor income	152 (54.9%)	65 (50.4%)	87 (58.8%)		
Medical insurance				1.984	0.159
Yes	259 (93.5%)	124 (96.1%)	135 (91.2%)		
No	18 (6.5%)	5 (3.9%)	13 (8.8%)		
Endowment insurance				3.794	0.051
Yes	160 (57.8%)	83 (64.3%)	77 (52.0%)		
No	117 (42.2%)	46 (35.7%)	71 (48.0%)		
Total score of sense of coherence, Median (Q1, Q3)	56 (49, 63)	60 (53, 70)	52 (47, 57)	14,275	*< 0.001*
Total score of illness perception, Median (Q1, Q3)	41 (34, 47)	35 (30, 43)	44.5 (41, 49)	4486.5	*< 0.001*
Confrontation, Median (Q1, Q3)	19 (17, 22)	19 (16, 22)	20 (18, 22)	8057	*0.025*
Avoidance, Median (Q1, Q3)	16 (14, 18)	15 (14, 18)	16 (14, 17)	8746.5	0.227
Acceptance-resignation, Median (Q1, Q3)	9 (6, 10)	7 (5, 9)	10 (8.75, 11)	4386.5	*< 0.001*
Total score of positivity, Median (Q1, Q3)	39 (37, 45)	39 (36, 44)	39.5 (38, 45)	8457	0.100

**Fig. 2:**
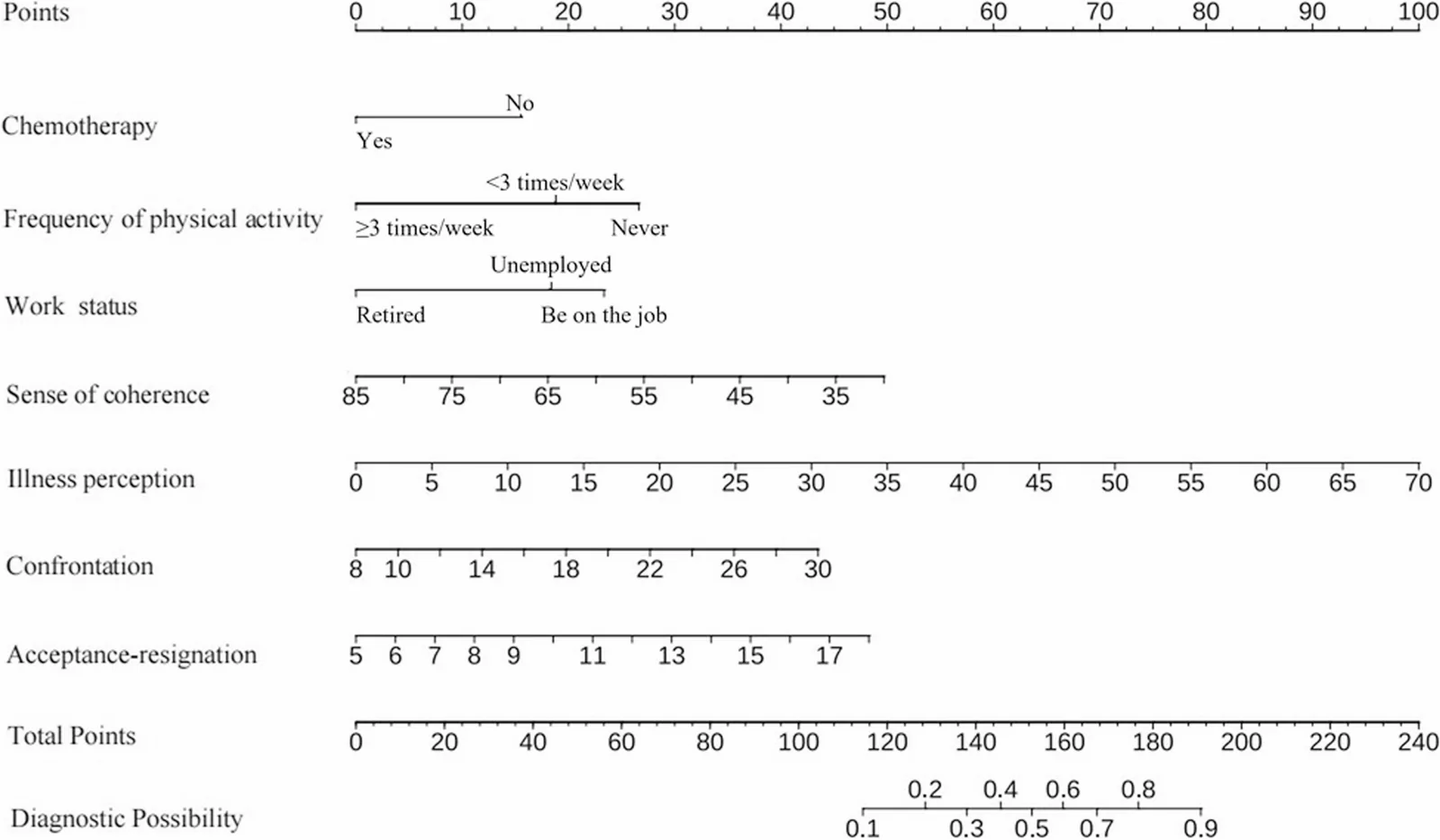
Nomogram for the prediction of FoP in postoperative patients with breast cancer

Following that, the backward election method using Akaike information criterion (AIC) was employed to identify the multivariable factors. Table 3 shows the final results. We found that chemotherapy (OR = 0.409, 95% CI: 0.145–1.066), frequency of physical activity [<3 times a week] (OR = 0.636, 95%CI: 0.302–1.322), frequency of physical activity [≥ 3 times a week] (OR = 0.216, 95% CI: 0.091–0.494), work status [unemployed] (OR = 0.744, 95% CI: 0.349–1.554), work status [retired] (OR = 0.261, 95% CI: 0.095–0.679), sense of coherence (OR = 0.949, 95% CI: 0.912–0.985), illness perception (OR = 1.086, 95% CI: 1.043–1.132), confrontation (OR = 1.12, 95% CI: 1.028–1.226), and acceptance-resignation (OR = 1.238, 95% CI: 1.064–1.450) exhibit the strongest association with psychological dysfunction in postoperative breast cancer patients. The independent influencing factors obtained from the multivariable analysis were utilized to construct a nomogram model for predicting psychological dysfunction in postoperative breast cancer patients, as presented in Fig. 2.

**Table 3:** Results of multivariable logistic regression analysis for fop in the training set

Variables	B	SE	OR	95% CI	Z	*P*
Constant	-1.684	1.939	0.186	0.003–8.336	-0.868	0.385
Chemotherapy	-0.894	0.504	0.409	0.145–1.066	-1.774	*0.076*
Frequency of physical activity [<3 times/week]	-0.453	0.375	0.636	0.302–1.322	-1.207	0.227
Frequency of physical activity [≥ 3 times/week]	-1.532	0.430	0.216	0.091–0.494	-3.562	*< 0.001*
Work status [unemployed]	-0.295	0.379	0.744	0.349–1.554	-0.778	0.436
Work status [retired]	-1.344	0.497	0.261	0.095–0.679	-2.703	*0.007*
Sense of coherence	-0.052	0.020	0.949	0.912–0.985	-2.649	*0.008*
Illness perception	0.082	0.021	1.086	1.043–1.132	3.978	*< 0.001*
Confrontation	0.114	0.045	1.120	1.028–1.226	2.551	*0.011*
Acceptance-resignation	0.214	0.079	1.238	1.064–1.450	2.717	*0.007*

### Validation and predictive accuracy of the nomogram model

**Fig. 3:**
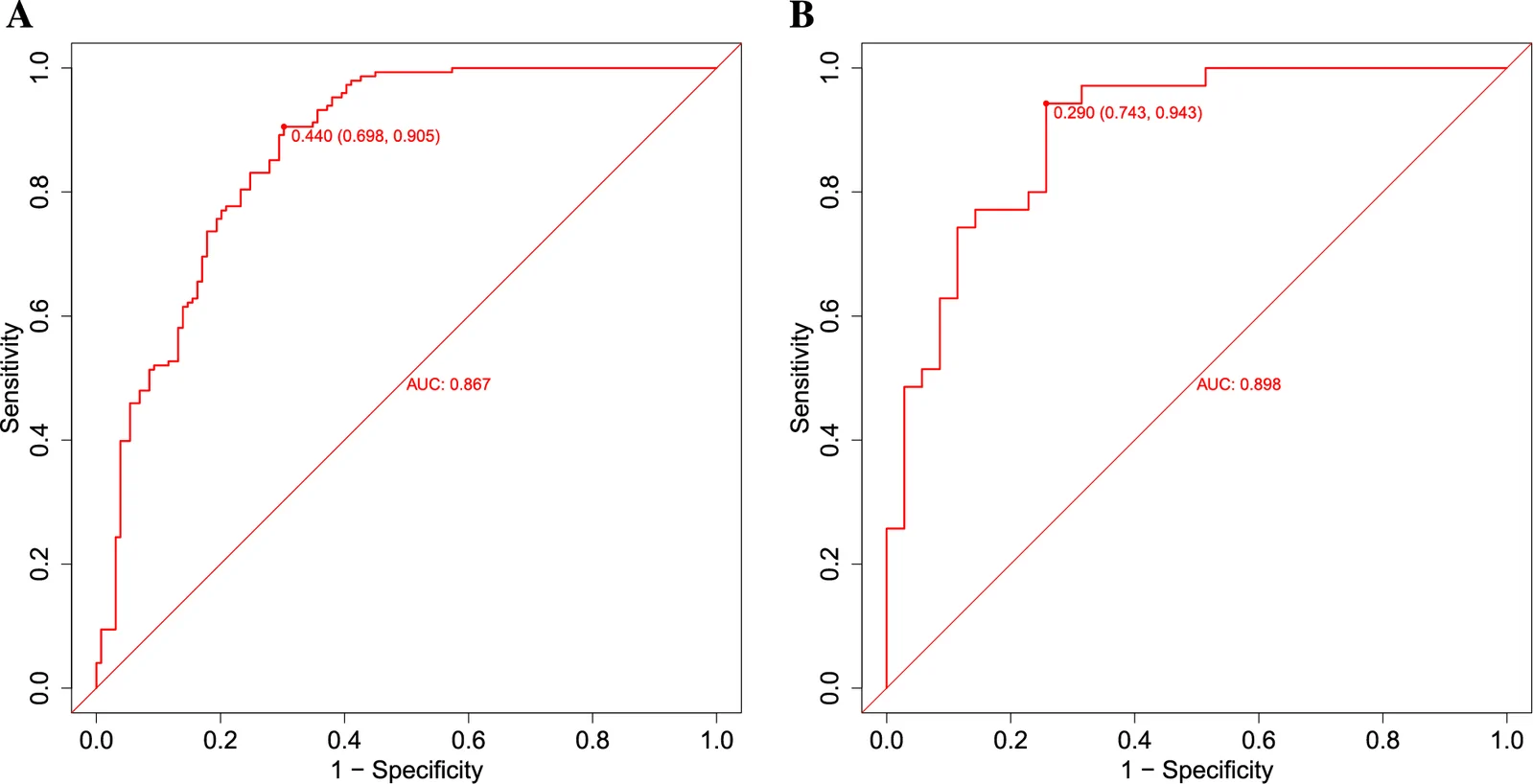
ROC curves of the nomogram in training set (**A**) and validation set (**B**)

**Fig. 4:**
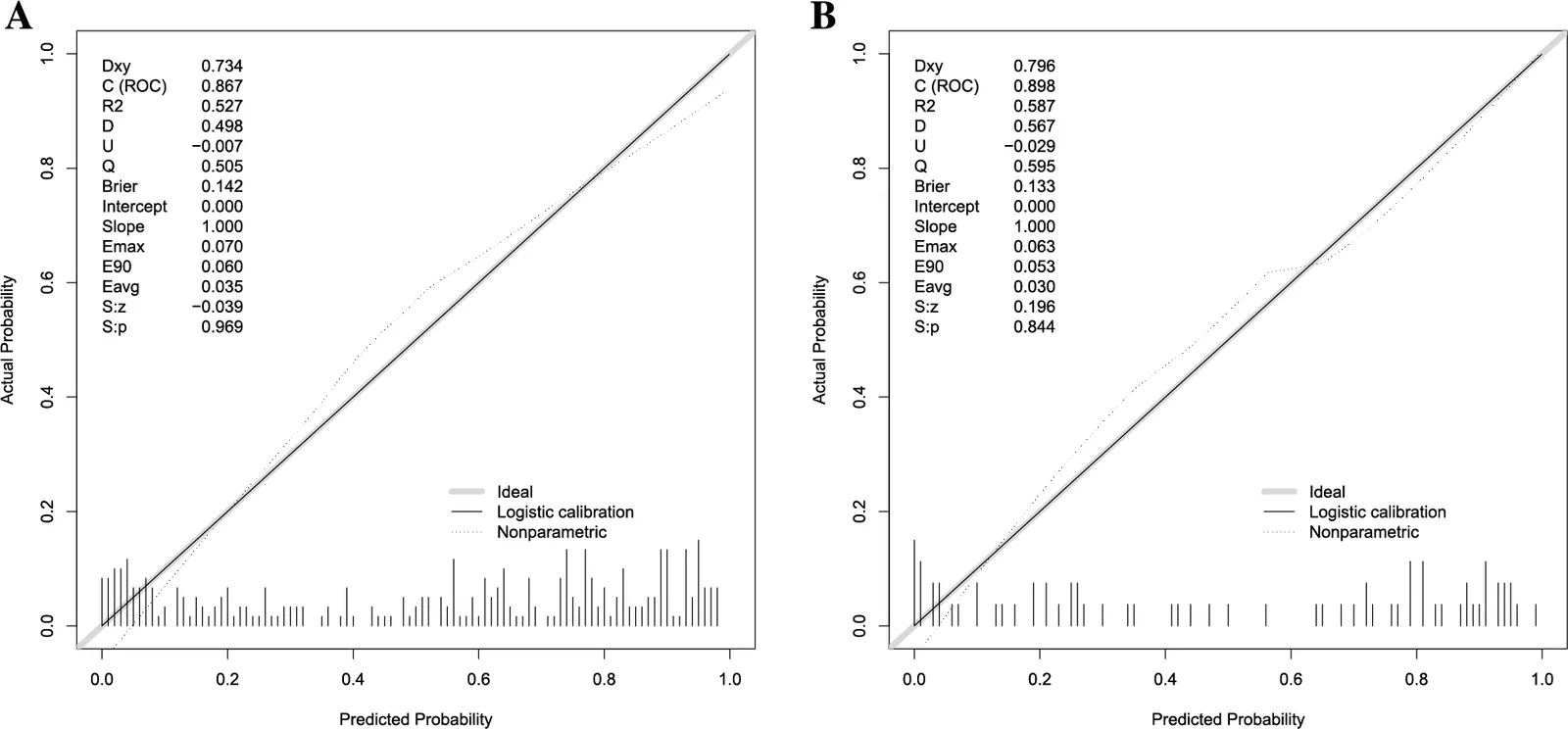
Calibration plots of the nomogram and actual values in the training set (**A**) and validation set (**B**)

**Fig. 5:**
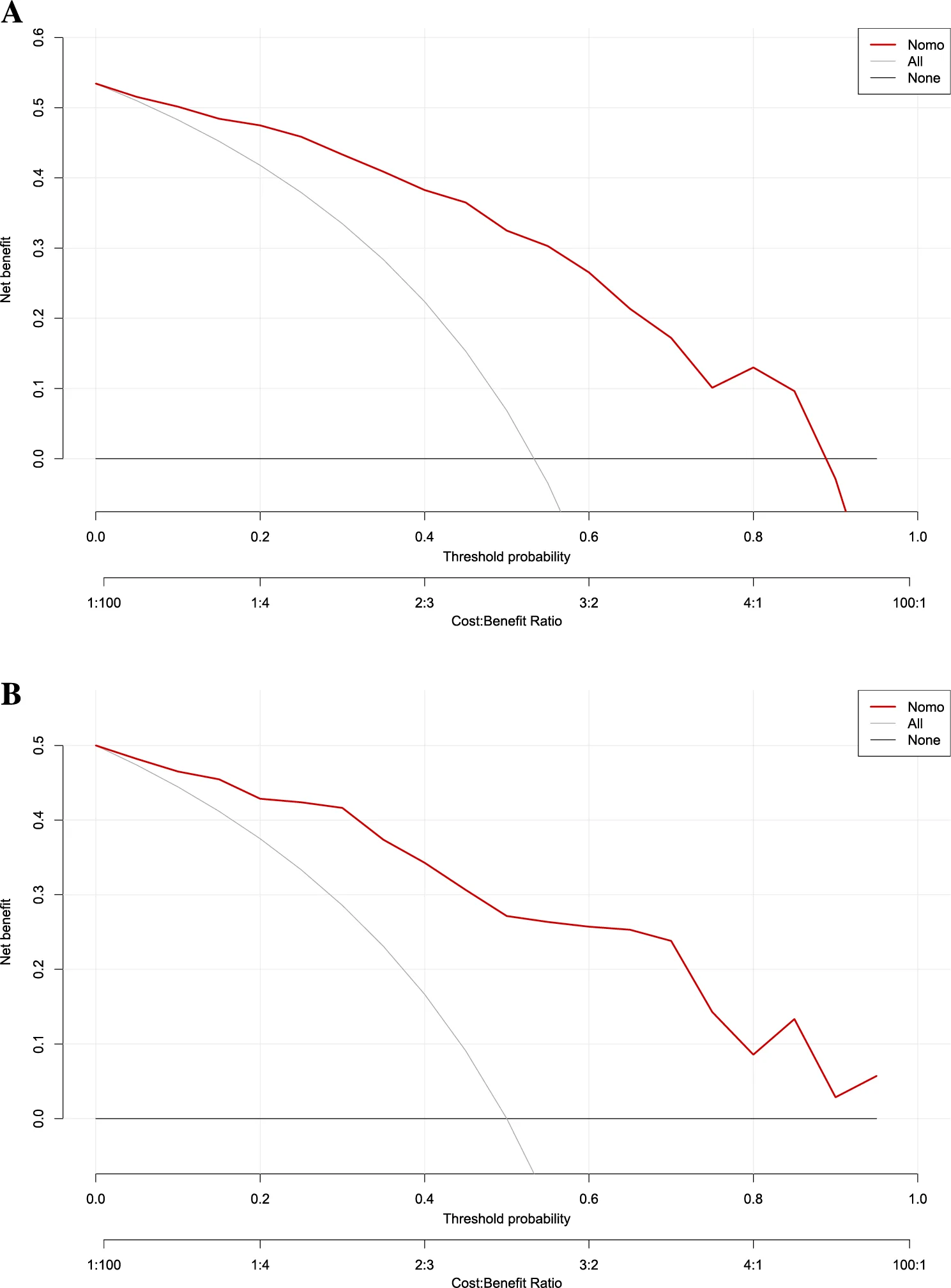
Decision curve analysis of the nomogram in training set (**A**) and validation set (**B**)

The nomogram model was validated and its predictive accuracy was assessed. The AUC in the training set was 0.867 (95% CI: 0.823–0.910), while in the validation set, that was 0.898 (95% CI: 0.826–0.970) (Fig. 3A-B). The calibration curve (Fig. 4A-B) represented by the Hosmer-Lemeshow *χ*^*2*^ statistic showed a good fit with a *P*-value of 0.086 (> 0.05) and a *χ*^2^ value of 13.837 for the training set, and a *P*-value of 0.246 (> 0.05) and *χ*^*2*^ value of 10.286 for the validation set. These results indicate that the nomogram demonstrated strong discriminative power in both sets of data analyzed. The DCA revealed that the training model exhibited a greater net advantage within the threshold probability range of 2%-92%, while the validation model demonstrated higher net utilization between the threshold probability intervals > 2% (Fig. 5A-B).

## Discussion

In this study, more than half of postoperative breast cancer patients experienced moderate to high level of FoP and showed psychological dysfunction, which is consistent to a previous study revealing that Chinese breast cancer patients experienced substantial FoP with an average score of 33.84 [15]. Based on the HEM framework, we found that variables related to individual traits (i.e., chemotherapy), as well as individual psychological and behavioral characteristics (i.e., physical activity, coping mode, illness perception, and sense of coherence) were independent predictors of high levels of Fop. This informed the development of the nomogram for predicting the risk of FoP among postoperative breast cancer patients, which could be a promotable tool for clinical practice.

Particularly, our findings revealed that patients who accepted chemotherapy after surgery tend to have lower levels of FoP, which is aligned with previous study suggesting cancer patients with psychological distress were less likely to have received chemotherapy [42]. Earlier evidence indicated that postoperative adjuvant chemotherapy reduced the risk of recurrence by 12.3% and mortality by 10% for patients with early-stage breast cancer at 15 years after surgery [43]. During the acute period after surgery and initiation of chemotherapy, patients may yield severe concerns about a variety of side effects on physical symptoms. With the reduction of its side effects over time, the administration of chemotherapy may enhance patients’ self-efficacy in disease management, as they observed clinical improvements and stabilization of their conditions, thereby reducing their symptom distress [44]. Therefore, healthcare providers should manage both physical and psychological effects during early period, particularly for patients who may be prone to heightened fear and anxiety. However, in this study, the proportion of participants who received chemotherapy was as high as 88.1%, while only 11.9% did not. This substantial imbalance may have biased the results toward the majority group, as the small sample size of the non-chemotherapy group limits the precision and stability of the estimated effect. Given the exploratory nature of this predictor within the prediction model, this finding should be interpreted with caution and warrants further validation in larger, more balanced samples.

Results showed that increased physical activity can significantly reduce the level of FoP among postoperative breast cancer patients. Evidence suggests that engaging in regular physical activity is associated with improved overall survival, reducing the risk of all-cause and breast cancer mortality by 42% and 40%, respectively [45]. These protective effects are likely due to the long-term regulation of various physiological factors, including sex hormone levels, insulin resistance, and inflammation [46]. However, a previous longitudinal study has highlighted a concerning trend of poor adherence to exercise regimens among postoperative patients, with a general decline in physical activity over time [47]. Recent data indicated that 64.7% of breast cancer survivors remained inactive even three years post-surgery [48]. Patients often perceived exercise as a physical burden and are more inclined to rest statically [49]. Besides, this lack of physical activity can be correlated with high levels of kinesiophobia, resulting in decreased musculoskeletal strength, weight gain, and a range of negative psychological outcomes [50]. To address these challenges, leveraging technology, such as wearable fitness trackers and mobile health apps, can motivate patients by tracking progress and providing real-time feedback. Additionally, CBT has demonstrated its statistically significant effects in reducing fatigue, improving quality of life and physical functioning [51], which is recommended to be combined with exercise interventions for cancer survivors by clinical practice guidelines [52].

Our study revealed that retired patients exhibited the lowest levels of FoP, whereas those who were employed or unemployed were more likely to develop FoP, consistent with previous research [53, 54]. Retired individuals often benefit from a stable psychological state, supported by consistent economic income, established social relationships, and access to a wealth of social resources, which largely contributes to their reduced FoP [55]. In contrast, individuals who are actively working face significant pressures in the workplace. Issues such as changes in physical appearance, adverse drug effects, sexual dysfunction, and early menopause resulting from surgery and postoperative adjuvant therapy can lead to psychological disturbances, including anxiety, sadness, and diminished self-esteem. Moreover, young and middle-aged women, who often balance critical family and social roles, encounter relatively unstable personal and professional environments. Their limited experience in managing multiple responsibilities, coupled with the potential impact of cancer on their future lives and careers, heightens their fear of cancer recurrence. Unemployed individuals, on the other hand, may experience more concern over the financial burden of treatment on their families, and may perceive their health condition as a barrier to their family’s well-being. These insights underscore the need for social support from primary caregivers and tailored psychological interventions that address the specific challenges faced by different demographic groups.

Sense of coherence refers to a pervasive, enduring though dynamic, feeling of confidence that one’s internal and external environments are predictable and that there is a high probability that things will work out as well as can reasonably be expected [36]. Studies have shown that cancer patients with higher sense of coherence were more adept at employing positive coping strategies, which significantly enhanced their psychological well-being [56, 57]. Our study further clarifies this relationship by demonstrating a negative association between the sense of coherence and FoP among postoperative breast cancer patients. Individuals with a strong sense of coherence are better equipped to comprehend their illness and treatment, manage stress, and exhibit a proactive readiness to face challenges. They are more likely to adopt effective coping strategies in response to environmental changes and actively engage in their treatment plans, which not only diminishes their fear of cancer recurrence but also reduces uncertainty about the disease [58]. To strengthen the sense of coherence, it is beneficial to encourage patients to engage in physical activities, mindfulness practices, and relaxation exercises, which can enhance their overall resilience and psychological stability, contributing to improved health outcomes [59].

Illness perception is a critical factor in how individuals understand and respond to their health conditions, shaped by the information they receive from healthcare professionals, family, and social networks. This perception profoundly impacts the way they manage their health and adhere to treatment [60, 61]. Our study identified a robust positive correlation between illness perception and FoP in postoperative breast cancer patients. Specifically, elevated illness perception scores were associated with an elevated sense of disease threat and heightened FoP, aligning with previous findings [62]. Patients who perceived their illness negatively often demonstrate increased sensitivity to physical symptoms and may overestimate the risk of recurrence, metastasis, or mortality. Such perceptions can detrimentally affect cognitive and social functioning, diminish quality of life, and lead to maladaptive coping strategies, thereby exacerbating fear and emotional distress [63, 64]. The development of illness perception is significantly influenced by personal experience and the information patients receive. Miscommunication or inadequate explanations regarding prognosis or treatment outcomes from healthcare providers or external sources can intensify patients’ sense of threat and vulnerability. To ensure patients have a comprehensive understanding of their condition, implementing psychoeducational programs through workshops, support groups, and informational sessions is essential to address common concerns and questions. Additionally, targeted psychological interventions, such as acceptance and commitment therapy (ACT), can assist patients in reframing negative thoughts and enhancing cognitive function, promoting a more balanced view of their illness [65]. In our study, we observed a statistically significant positive correlation between FoP and acceptance-resignation among postoperative breast cancer patients, aligning with the previous findings [66]. Patients who perceived their control over the disease as limited may feel vulnerable to the uncertainties of their health status. This may result in decreased adherence to treatment and lack of proactive engagement in managing their health, which could in turn exacerbate feelings of helplessness and anxiety about the future. Interestingly, our study revealed elevated FoP levels in patients who used confrontational coping strategies, in contrast to previous findings that active coping generally mitigates the psychological impact of adverse events [66]. Several mechanisms may account for this discrepancy. First, patients who adopt a confrontational approach often actively seek out health-related information during treatment and recovery; however, the large volume of unverified, conflicting, or overly alarming content, particularly from online sources, may inadvertently intensify perceptions of disease risk, leading to heightened anxiety and depression rather than perceived control [24]. Second, limited health literacy may further compound this issue. Previous research suggests that patients with inadequate health literacy are more likely to misinterpret information and be influenced by biased sources, which can contribute to increased distress, poorer functional status, and greater healthcare utilization [67]. Third, confrontation coping may be misdirected in some patients, manifesting as repetitive reassurance-seeking, excessive monitoring of physical symptoms, or passive consumption of unreliable information without effective processing [68]. Such maladaptive patterns may paradoxically amplify fear of progression rather than alleviate it. Compounding this issue is the inherent uncertainty of clinical practice, which permeates all aspects of care, including diagnosis, treatment, prognosis, relationship-building, and emotional experiences. Clinicians almost invariably use language expressing uncertainty when explaining their diagnostic reasoning, yet such uncertainty itself may provoke negative emotional responses in patients, including anxiety, denial, aversion, or inaction [69]. When patients are exposed to this uncertainty without effective coping strategies and without adequate guidance from healthcare professionals, their maladaptive behaviors may be reinforced, thereby creating a vicious cycle. In this case, healthcare providers should prioritize clear and effective communication, using plain language and visual aids to help patients understand their disease progression and treatment. Additionally, incorporating health literacy assessments into routine care can identify patients who may need additional support, while integrating digital tools and resources can provide accessible information outside of clinical settings.

### Limitations

To the best of our knowledge, this is the first study developing and evaluating a user-friendly and relatively personalized model guided by HEM to predict the risk of FoP among postoperative breast cancer patients. The constructed nomogram provided an easy-to-use and individualized model for prediction of FoP risk in breast cancer patients after surgery, which is helpful for optimizing clinical management. The ROC curve, calibration plots and DCA curves suggest that the model has good discrimination, calibration, and clinical benefit. However, some limitations in our study should be acknowledged. First, the outcomes were self-reported, which may lead to a bias of over- or under-estimation of the actual measurement for FoP. Second, the interpretation of the association between chemotherapy and FoP is limited by the substantial imbalance in sample size (88.1% vs. 11.9%), which may compromise the reliability and generalizability of this finding. Third, external validation of the nomogram was not performed owing to constraints of time and resources. Although the model exhibited favorable performance in internal validation, its applicability to other populations remains uncertain. Independent external validation is necessary to establish the robustness and reproducibility of the predictive model prior to its clinical implementation. Finally, despite the multicenter design, all participating institutions were located within a single province. This geographic limitation may introduce regional bias, as patient characteristics, healthcare delivery, and sociocultural contexts can vary considerably across regions. Consequently, the representativeness of our sample and the generalizability of the findings to broader populations may be constrained. Future research should prioritize validating the nomogram across geographically diverse settings, utilizing larger sample sizes and extended follow-up periods to strengthen and expand the applicability of our results.

## Conclusions

This study developed a HEM-based predictive model, where we identified chemotherapy, frequency of physical activity, work status, sense of coherence, illness perception, coping mode as predictors for the risk of FoP among postoperative breast cancer patients. The model showed good calibration capability. The resulting nomogram can be used to assist clinical practitioners to identify risk of FoP, thereby develop personalized care program tailored for postoperative breast cancer patients to mitigate the level of FoP.

## Data Availability

The raw data supporting the conclusions of this article will be made available by the authors, without undue reservation.

## Abbreviations

AUC: Area under the curve
AIC: Akaike information criterion
CBT: Cognitive behavior therapy
DCA: Decision curve analysis
FoP-Q-SF: Fear of Progression Questionnaire-Short Form
HEM: Health ecology model
MBSR: Mindfulness-based stress reduction
ROC: Receiver operating characteristic
SOC-13: Sense of Coherence-13
